# Mechanisms and advantages of natural derived small molecule compounds in the prevention and treatment of colorectal cancer: a review

**DOI:** 10.3389/fphar.2025.1658493

**Published:** 2025-09-10

**Authors:** Ming-Jie Liao, Hao-Yu Dong, Gang Chen, Wei-Wei Li, Guo-Feng Li

**Affiliations:** Shenzhen Baoan Authentic TCM Therapy Hospital, Shenzhen, China

**Keywords:** colorectal cancer, natural derived small molecule compounds, mechanism of action, gut microbiota, ferroptosis, apoptosis, autophagy

## Abstract

Globally, colorectal cancer (CRC) ranked third in cancer prevalence and emerged as the primary contributor to cancer-related fatalities in 2022, with projections indicating substantial escalation by 2040. The malignant progression of healthy colonic cells involves complex interactions among multiple cellular pathways over extended periods (typically exceeding 10 years), influenced by dietary patterns, lifestyle factors, and genetic predispositions. In addition, marked disparities in CRC incidence and mortality appear to show large differences across geographic regions, demographic groups, and biological sexes, suggesting that there are traces of CRC. Therefore, timely intervention or regression of the development of CRC, particularly targeting high-risk populations, may be an excellent strategy to reduce CRC burden in forthcoming decades. Natural derived small molecule compounds (NDSMCs) exhibit significant advantages, including structural diversity, unique biological activities, low toxicity and multi-target effects. Increasing evidence suggests that NDSMCs demonstrate therapeutic potential against CRC through multi-target mechanisms, such as modulation of gut microbiota, induction of ferroptosis, and regulation of programmed cell death pathways (apoptosis/autophagy), thereby offering promising avenues for CRC treatment. However, comprehensive reviews in this field remain scarce. Consequently, this study systematically summarizes the research advancements over the past 5 years regarding the mechanisms of NDSMCs in combating CRC, aiming to provide valuable insights for therapeutic strategies, preventive measures, and novel drug development. Furthermore, the clinical progress and limitations of certain NDSMCs in CRC treatment are also discussed.

## Graphical Abstract

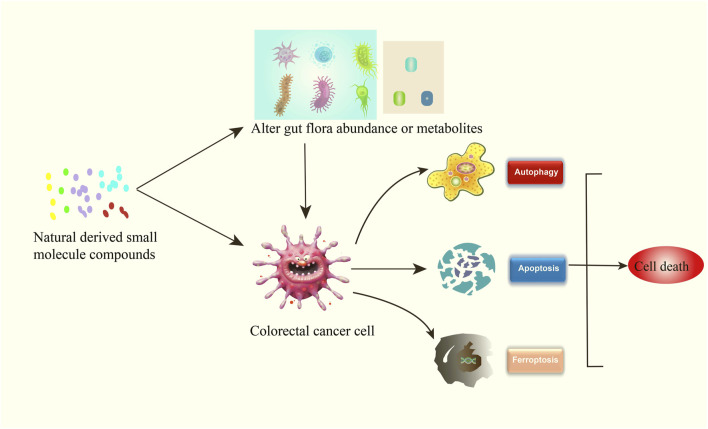

## 1 Introduction

Colorectal cancer (CRC) is the third most common malignancy and a major contributor to cancer-related mortality worldwide (Wu et al., 2025). Global estimates for 2020 indicated over 1.9 million newly reported CRC cases alongside approximately 930,000 fatalities attributed to this disease (Morgan et al., 2023). Projections suggest a good deal of escalation in CRC prevalence by 2040, with anticipated annual figures reaching 3.2 million incident cases (marking a 63% surge) and 1.6 million deaths (reflecting a 73% rise) (Olfatifar et al., 2025).

**FIGURE 1:**
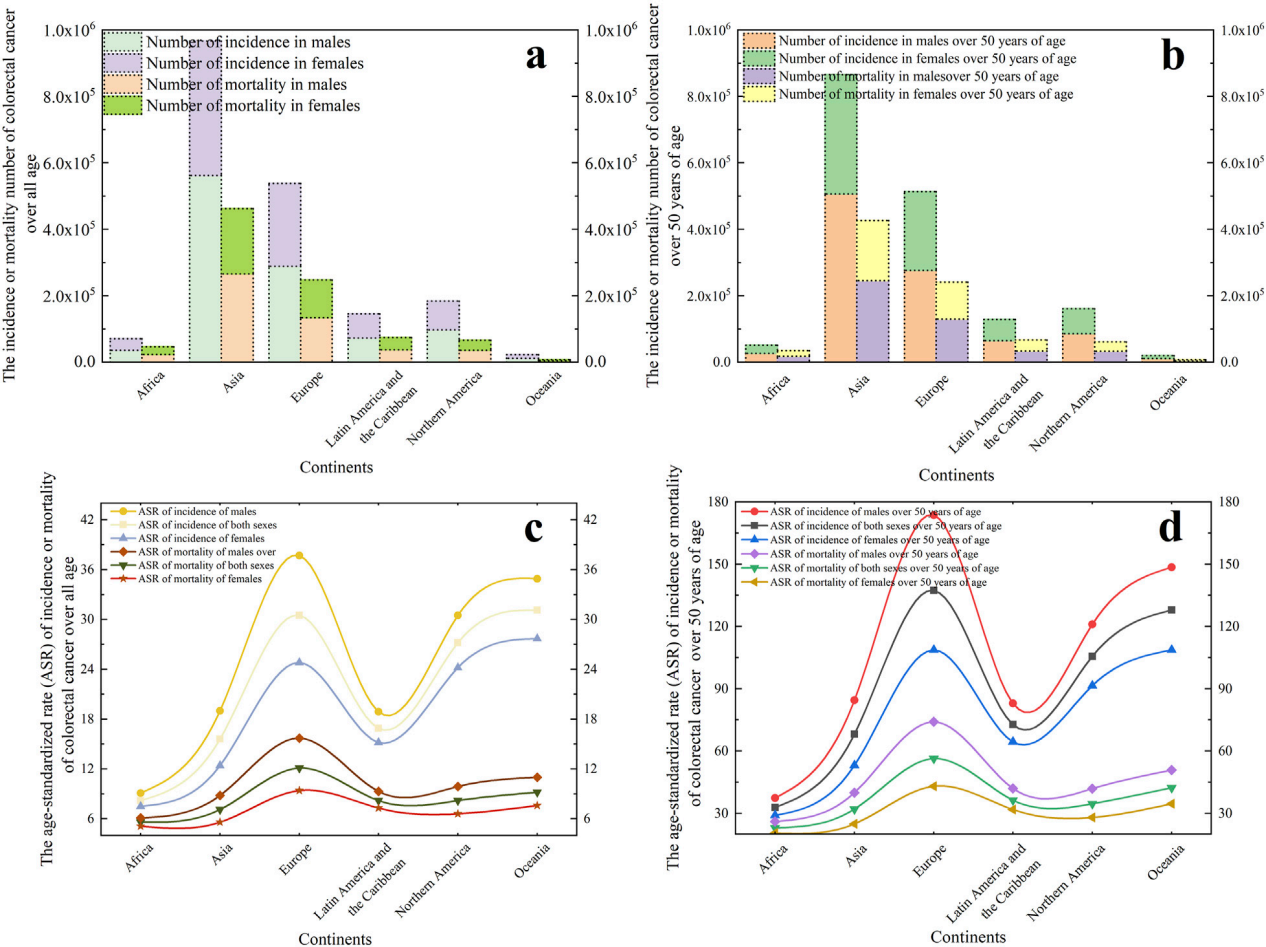
In 2022, global colorectal cancer statistics across six continents, presenting case counts and age-standardized rates (ASR) for both incidence and mortality. **(a)** The incidence or mortality number of colorectal cancer over all age. **(b)** The incidence or mortality number of colorectal cancer r focusing on individuals over 50 years old. **(c)** The ASR of incidence or mortality of colorectal cancer over all age. **(d)** The ASR of incidence or mortality of colorectal cancer focusing on individuals over 50 years old.

The incidence and mortality of CRC appear to exhibit significant disparities across gender, geographic regions, and age groups, suggesting that CRC incidence has a track record. Data (Figure 1) from https://gco.iarc.fr/today/en/dataviz reveal Asian and European populations collectively responsible for 78.10% of global cases and 78.55% of mortality figures. Given substantial demographic variations between these regions, age-standardized rates (ASR) per 100,000 population provide more accurate comparisons of CRC burden. This statistical method normalizes population age structures, enabling meaningful cross-regional analysis. Epidemiologic data demonstrates striking continental variations, with Oceania (31.1), Europe (30.5), and North America (27.2) showing incidence ASRs 2-3 times higher than other continents. Mortality patterns mirror this distribution, with Europe demonstrating the highest mortality ASR at 12.1, followed by Oceania (9.2) and North America (8.2). For individuals aged above 50 years, Oceania exhibited the highest age-standardized incidence rates (137.4), followed by Europe (127.9) and North America (105.6). Mortality patterns showed Europe leading with 56.4 age-standardized mortality per 100,000 population, trailed by Oceania (42.3) and Latin America (36.4). Notably, over nine-tenths of total CRC cases and fatalities occurred in this older demographic cohort. In addition, male populations demonstrated 24.79% higher incidence rates and 23.63% elevated mortality compared to females.

The observed variations primarily stem from CRC’s multifactorial etiology, which encompasses hereditary predisposition, dietary patterns, and lifestyle choice (Cañellas-Socias et al., 2024). From a genetic perspective, individuals with familial histories of colorectal polyps-including specific syndromes like familial adenomatous polyposis, Lynch syndrome and mutY DNA glycosylase-associated polyposis-demonstrate elevated susceptibility to CRC development. Nutritional and behavioral factors such as insufficient dietary fiber consumption coupled with excessive consumption of animal-derived proteins and lipids, along with sedentary behaviors, tobacco use, and chronic alcohol abuse, collectively contribute to heightened disease risk (Chan et al., 2025). Furthermore, chronic gastrointestinal inflammatory conditions including persistent ulcerative colitis, Crohn’s disease, and other prolonged inflammatory states have been clinically identified as significant risk factors for colorectal carcinogenesis (Shahgoli et al., 2024).

The progression from healthy colonic epithelial cells to advanced adenocarcinomas typically spans over 10 years, involving complex interactions among multiple biological mechanisms that respond to nutritional factors, behavioral patterns, and genetic pressures, which process evolves through precancerous adenomatous polyps before manifesting the morphological and functional transformations characteristic of malignant tumors (Zheng et al., 2023). Implementing intervention or regression (prevention) of CRC development timely, especially targeting key areas, age ranges, and genders, could emerge as a promising approach for substantially decreasing CRC-related incidence and mortality in forthcoming years.

Contemporary clinical management of CRC primarily involves multimodal therapeutic approaches including surgical intervention, radiation treatment, systemic chemotherapy, combination regimen (Kumar et al., 2023) or molecularly targeted agents (Zheng et al., 2023; Feng et al., 2024). Despite these interventions, patient outcomes remain suboptimal, with survival statistics demonstrating marked disparity across disease stages. Epidemiological data reveals stage I CRC patients achieve approximately 90% 5-year survival probability, contrasting sharply with the dismal 10% survival rate observed in stage IV cases, underscoring the limitations of existing therapeutic paradigms (Xie et al., 2020). Furthermore, chronic treatment administration imposes substantial socioeconomic burdens while inducing multisystem adverse effects spanning gastrointestinal disturbances (nausea, emesis, diarrheal episodes), mucosal complications (oral ulceration), organ-specific toxicities (hepatic impairment), and hematological sequelae including myelosuppression and immune dysfunction (Miao et al., 2025).

**FIGURE 2:**
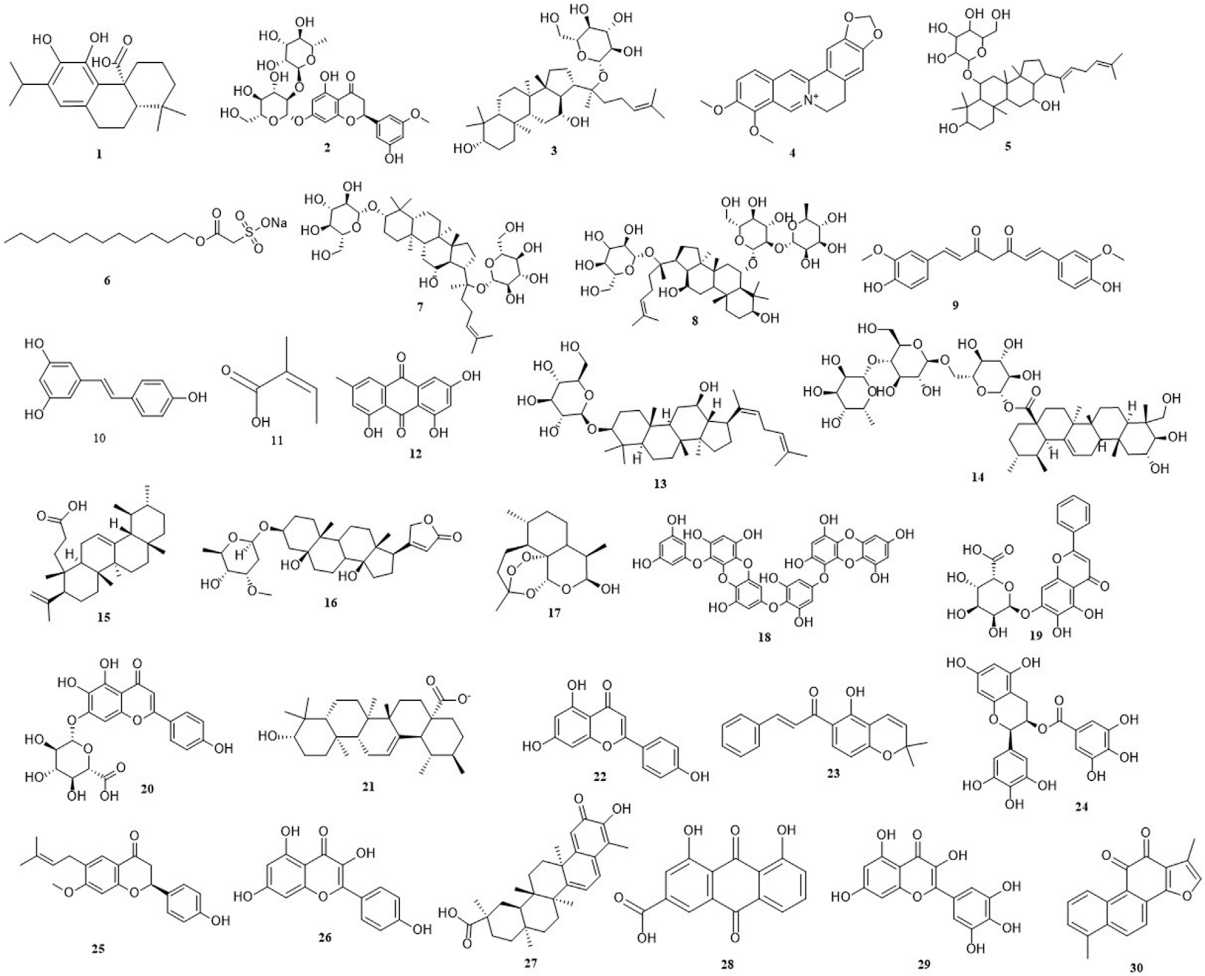
The chemical structures of all the natural derived small molecule compounds mentioned in this article. (**1**) Carnosic acid. (**2**) Neohesperidin. (**3**) Ginsenoside compound K. (**4**) Berberine. (**5**) Rare ginsenoside Rh4. (**6**) New Houttuynia sodium folate. (**7**) Ginsenoside-F2. (**8**) Ginsenoside-Re. (**9**) Curcumin. (**10**) Resveratrol. (**11**) Angelic acid. (**12**) Emodin. (**13**) Ginsenoside Rh3. (**14**) Asiaticoside. (**15**) Roburic acid. (**16**) Periplocymarin. (**17**) Dihydroartemisinin. (**18**) Dieckol. (**19**) Baicalin. (**20**) Scutellarin. (**21**) Ursolic acid. (**22**) Apigenin. (**23**) Lonchocarpin. (**24**) (−)-Epigallocatechin-3-Gallate. (**25**) Bavachin. (**26**) Kaempferol. (**27**) Celastrol. (**28**) Rhein. (**29**) Myricetin. (**30**) Tanshinone.

During recent years, a growing number of studies have confirmed that natural derived small molecular compounds (NDSMCs) exhibit multiple mechanisms of action in the prevention and treatment CRC by regulating the intestinal microbiota (Lu et al., 2022; Weng and Goel, 2022), ferroptosis (Wu et al., 2023a), apoptosis (Li et al., 2023), autophagy (Secme et al., 2023) by not only stopping cancer progression or reversing carcinogenic effects in the precancerous stage, but also significantly restrain the proliferation activity of CRC cells, induce programmed death, hinder the metastasis process and inhibit angiogenesis. Therefore, this study systematically summarizes the research progress of NDSMCs in the fight against CRC in the past 5 years, including intestinal microbiota regulation, ferroptosis induction, apoptosis activation and autophagy regulation, to establish causation theories for clinical treatment strategy optimization and innovative drug development. In addition, clinical advances and shortcomings in the treatment of CRC with some NDSMCs have also been reported. The chemical structures of all the NDSMCs mentioned in this article has been shown in Figure 2.

## 2 Natural derived small molecule compounds treat colorectal cancer by regulating intestinal flora composition and metabolites

The gut microbiota, comprising diverse microbial populations inhabiting the human digestive system’s gastrointestinal tract, undergoes dynamic modulation through various determinants including host genetics, aging processes, nutritional patterns, daily habits, pathological conditions, and pharmacological interventions (Barko et al., 2018; Ma et al., 2022). A well-regulated gut microbial ecosystem enhances immune function and inhibits CRC progression, whereas microbial dysbiosis characterized by pathogenic bacterial overgrowth facilitates CRC initiation and advancement (Wang J. et al., 2024). In recent years, numerous reports have indicated that NDSMCs exert significant anti-CRC effects by interacting with the intestinal microbiota to alter the abundance of the overall microbiota or specific bacteria, or influencing microbiota-derived metabolic byproducts (Table 1). Of course, gut microbiota actively participating in modifying NDSMCs' biotransformation processes and nutrient assimilation, consequently impacting their anticancer efficacy (Fujisaka et al., 2023).

**TABLE 1:** Natural derived small molecule compounds treat and improve colorectal cancer by regulating intestinal microbiota and its mechanism of action.

Compounds	Model	Changes in the gut microbiota	Related mechanism	References
Carnosic acid	JGpt-Apcem1Cin(min)/Gpt (ApcMin/+) mice	↓*Bacteroide*, ↓*Bifidobacterium*, ↓*Alistipe,* ↓*Desulfovibrio,* ↑*Faecalibacterium,* ↑*Subdoligranulum*	↓NF-κB/STAT3 signaling, ↓IL-1β, ↓IL-6, ↓CXCL1, ↓IL-17, ↓DL-lactate, ↓citric acid	Li et al. (2022)
Neohesperidin	APC (min/+) transgenic mouse model	↓*Bacteroidetes*, ↑*Firmicutes*, ↑ *Proteobacteria*	—	Gong et al. (2019)
Ginsenoside compound K	HCT-116, HT-29 and LOVO cells	↑*Akkermansia spp*., ↓Rikenellaceae*_RC9_gut_group*	—	Shao et al. (2022)
Berberine	AOM/dss-induced CRC model mice fed with HFD	↑*Akkermansia*, ↑*Parabacteroides*	↓IL-6/STAT3, ↓Wnt signaling pathways	Chen et al. (2023)
Rare ginsenoside Rh4	AOM/DSS-induced mouse model of CRC	↑*Akkermansia muciniphila*	↑7α-hydroxy-steroid dehydrogenase activity, ↑UDCA ↑FXR, ↓ TLR4-NF-κB signaling pathway	Bai et al. (2024)
New Houttuynia sodium folate	*Fn* load in the CRC-cells-derived mice xenografts	↓*F. nucleatum*	↓FadA, ↓cancer-driven inflammation	Jia et al. (2022)
Panax notoginseng saponin	AOM/DSS induced colon tumorigenesis mouse	↑*Akkermansia spp*	↑the richness and diversity of intestinal flora	Chen et al. (2020)
Pien Tze Huang	AOM/dss-induced CRC model mice	↑*Pseudobutyrivibrio xylanivorans*, ↑*Eubacterium* ↓*limosum*, ↓*Aeromonas veronii*, ↓*Campylobacter jejuni*, ↓*Collinsella aerofaciens*, ↓ *Peptoniphilus harei*	↑taurine, ↑hypotaurine, ↑bile acids, ↑ unsaturated fatty acids, ↓IL-17, ↓TNF, ↓ cytokine-chemokine	Gou et al. (2023)
Curcumin	The AOM/DSS-induced CRC model mice	↓*Ileibacterium, Monoglobus*, ↓*Desulfovibri*, ↑ *Clostridia_UCG-014*, ↑*Bifidobacterium*, ↑ *Lactobacillus*	↑the richness and diversity of intestinal flora	Deng et al. (2024)

Some bacterial species (*B. fragilis*, *Fusobacterium nucleatum* and *Porphyromonas asaccharolytica*)) demonstrate higher abundance in CRC and the classified as detrimental microorganisms (Torres-Maravilla et al., 2021), conversely, microbial strains including *Clostridium butyricum*, *Streptococcus thermophilus*, and *Lacticaseibacillus paracasei* exhibit protective effects against CRC development (Doo et al., 2024). A primary mechanism through which NDSMCs counteract CRC progression involves mitigating inflammatory processes and immune activation triggered by pathogenic bacterial interactions with host tissues (Yang and Cong, 2021). For example, the components or secretions of certain intestinal microorganisms, such as lipopolysaccharides (LPS), short-chain fatty acids (SCFAs) (Hou et al., 2022), probile acids (Kuhls et al., 2022) and azoxymethane (Dzhalilova et al., 2023), have been identified as mediators of chronic intestinal inflammation and immune dysregulation, significantly contributing to CRC pathogenesis, while other metabolites, including ursodeoxycholic acid (UDCA) (Zhang et al., 2021a) and butyrate (Mann et al., 2024), exhibit therapeutic advantages and counteract tumorigenesis and disease progression.

Here, a brief introduction to the effects of some star molecules on CRC were introduced. SCFAs, microbial-derived metabolites synthesized through bacterial breakdown of dietary fibers in anaerobic intestinal environment (Liu Y. et al., 2023). The three predominant SCFAs, butyrate, propionate and acetate, exhibit protective properties against CRC through distinct mechanisms (Blaak et al., 2020). Butyrate serves as the primary energy substrate for colonic epithelial cells, enhancing cellular differentiation processes while mitigating inflammatory responses, which barrier-strengthening effects manifest through upregulated tight junction protein synthesis, stimulated mucin 2 secretion, and HIF-1 pathway stabilization (Zhang et al., 2025). Propionate can inhibit malignant transformation and induces apoptosis in precancerous CRC, although it is less effective (compared with butyrate) and less abundant in CRC (Cheong and Trefely, 2025). Acetate undergoes metabolic conversion by specific colonic microbiota including *Roseburia* spp., *Faecalibacterium prausnitzii*, and *Coprococcus* species, functioning as the terminal product in this fermentation cascade and concentrated to butyrate by the action of butyryl-coa: acetate transferase (Hosmer et al., 2024). Elevated lactate levels within tissue microenvironments serve as a hallmark of neoplastic progression and inflammatory conditions (Dai et al., 2024). Citrate present in extracellular spaces may act as a danger-associated molecular pattern, stimulating inflammatory responses via NOD-like receptor protein 3 (NLRP3) inflammasome activation and subsequent nuclear translocation of nuclear factor kappa-B (NF-κB) (Parkinson et al., 2021). The bidirectional relationship between bile acid metabolism and gut microbiota exerts both protective and pathogenic influences on host physiology (Collins et al., 2023). Specific bile acids including Cholic acid (CA) and chenodeoxycholic acid (CDCA) demonstrate carcinogenic potential in CRC through modulation of NF-*κ*B and JAK2/STAT3 signaling cascade (Fuchs and Trauner, 2022). Conversely, UDCA exhibits preventive properties by activating farnesoid X receptor signaling while suppressing TLR4-mediated pathways.

In addition, reducing the production of metabolites that promote CRC development and elevating beneficial metabolites is another important mechanism (González et al., 2024). As a recognized contributor to CRC, *F. nucleatum* secretes the virulence factor FadA that interacts with E-cadherin on intestinal epithelial cells, activating the Wnt-β-catenin signaling cascade which upregulates cyclin D1 expression and accelerates cellular proliferation (Ou et al., 2022). The bacterial component LPS additionally stimulates Toll-like receptor 4 (TLR4) receptors, initiating MYD88-dependent NF-κB activation that elevates miRNA-21 levels while suppressing RASA1 expression and molecular cascade subsequently triggers RAS-MAPK signaling, leading to S-phase cell cycle arrest and consequent enhancement of malignant cell multiplication in CRC (Sulit et al., 2023).

Carnosic acid (Figure 2, **1**), a phenolic diterpene predominantly extracted from rosemary, exhibits notable anti-inflammatory characteristic (Li et al., 2022; Li et al., 2022) found that the abundance of *Bacteroides, Bifidobacterium, Alistipes* and *Desulfovibrio* were downregulated by carnosic acid, while enhancing beneficial anti-inflammatory genera such as *Faecalibacterium* and *Subdoligranulum*, which relies on the degradation of acetic acid to produce butyric acid and anti-inflammatory properties by effectively control pro-inflammatory cytokines IL-1β, IL-6, CXCL1, and IL-17, thus showing anti-CRC properties, particularly, *Faecalibacterium*, which further reduces the production of DL-lactate and citric acid.

Neohesperidin (Figure 2, **2**) derived from citrus fruits was discovered by Akhter et al. (2024) that the CRC would be inhibited and angiogenesis would be blocked by neohesperidin. However, interestingly, subsequent investigations demonstrated that neohesperidin’s protective effects operate independently of direct tumor cell interaction or Wnt/β-catenin pathway modulation, which is through microbial population shifts, specifically decreasing *Bacteroidetes* while elevating *Firmicutes* and *Proteobacteria* abundance at the phylum level. Instead, it exerts its anti-CRC effect by reducing, at the phylum level, the relative abundance of *Bacteroidetes* and increasing the abundances of *Firmicutes* and *Proteobacteria*. Critical validation through fecal microbiota transfer trials and antibiotic-mediated reversal of neohesperidin’s CRC suppression conclusively established gut microbiome modification as the principal mechanism underlying neohesperidin’s preventive action against colorectal tumor formation.

**FIGURE 3:**
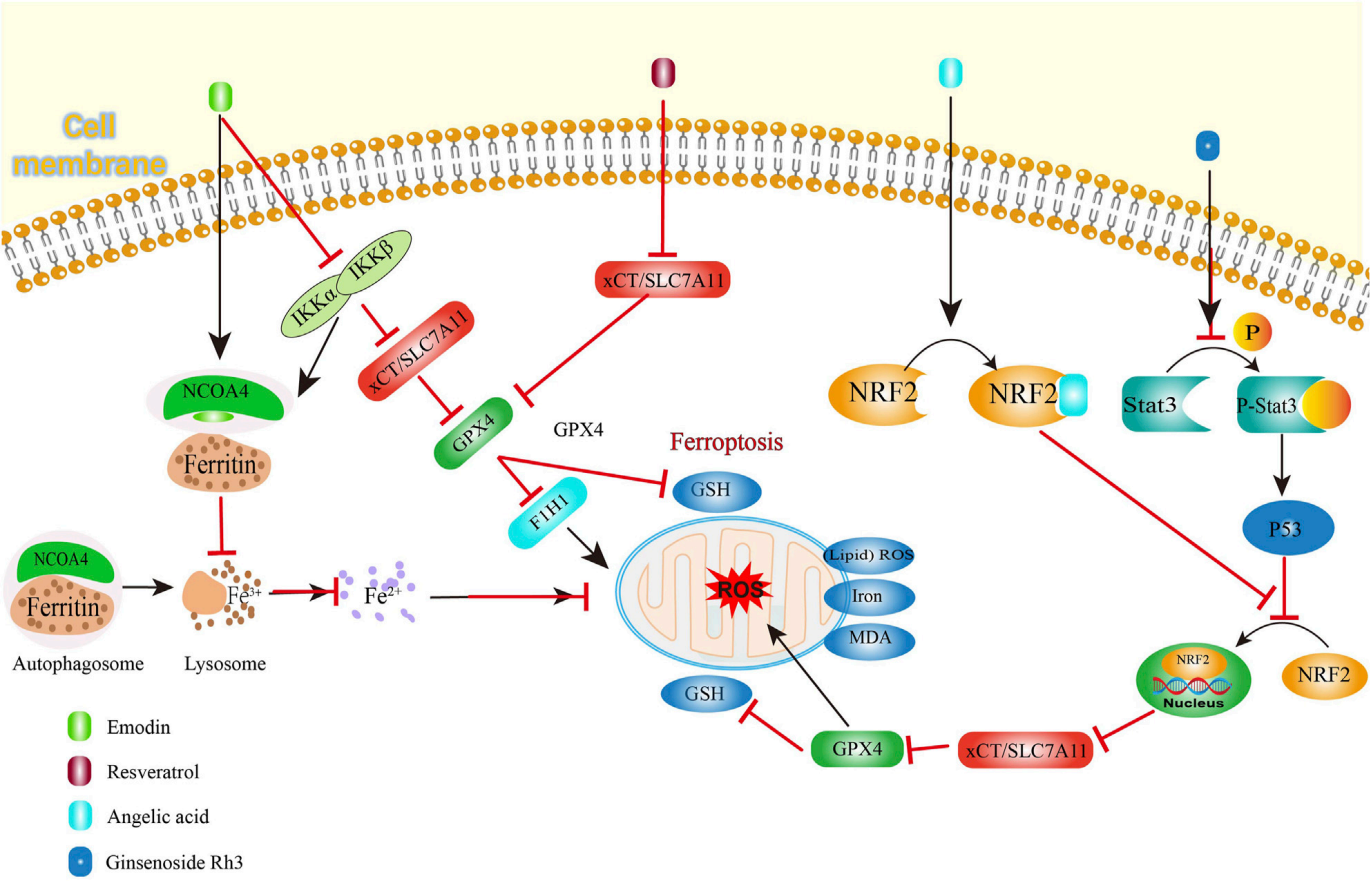
Natural derived small molecule compounds treat colorectal cancer by promoting ferroptosis.

Ginsenoside compound K (Figure 2, **3**), a dammarane-type tetracyclic triterpene, is derived from *Panax ginseng*, demonstrating diverse therapeutic properties including hypoglycemic, anti-aging, anti-allergic, anti-inflammatory and CRC-suppressing activities (Shao et al., 2022; Shao et al., 2022) found that ginsenoside compound K exerts anti-CRC effects through gut microbiota modulation, specifically enhancing populations of *Akkermansia* species (mucin-degrading commensals) while suppressing pathogenic Rikenellaceae_RC9_gut_group populations in AOM/DSS-induced colitis models.

**FIGURE 4:**
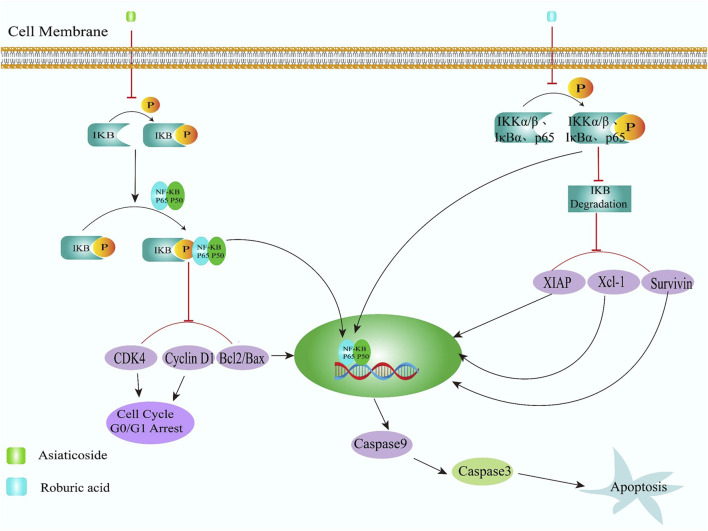
Natural derived small molecules induce apoptosis of colorectal cancer cells by inhibiting nuclear factor kappa-beta (NF-κB) signaling pathway.

High-fat diet (HFD) is positively correlated with the risk of CRC (Tang et al., 2024). Berberine (BBR) (Figure 2, **4**), a bioactive isoquinoline alkaloid derived from Coptis chinensis, exhibits antimicrobial properties and has been widely employed in managing gastrointestinal infections including bacterial enteritis and dysentery (Huang and Huang, 2025; Chen et al., 2023) demonstrated that BBR inhibits CRC progression in HFD-fed mice with AOM/DSS-induced carcinogenesis through dual pathway modulation (IL-6/STAT3 and Wnt signaling pathways) thereby suppressing cell proliferation by increasing the abundance of beneficial gut microbiota, including *Akkermansia* and *Parabacteroides* in mice with AOM/DSS-induced CRC models fed with HFD. At the same time, BBR treatment notably decreased the incidence of colonic polyps while restoring intestinal barrier integrity and ameliorating microbial imbalance. In addition, glycerophospholipid metabolism is often significantly altered in the progression of CRC associated with HFD in mice. In this study, BBR treatment was found can reverse these changes in glycerophospholipid metabolites, especially reducing the concentration of lysophosphatidylcholine, proven to stimulate CRC cell growth and exacerbate cellular junction impairments. Notably, the anti-tumor efficacy of BBR was not observed in CRC models with depleted intestinal flora from HFD-fed mice, though transplanting the gut microbiota from BBR-treated mice via fecal microbiota transplantation restored the tumor-suppressing effects on both CRC development and lysophosphatidylcholine regulation, emphasizing gut microbiome’s crucial role in countering HFD-induced CRC. In AOM/DSS-induced CRC models, BBR administration enhanced the abundance of SCFA-producing bacteria including Prevotellaceae and *Alloprevotella*, consequently elevating concentrations of acetate, propionate, and butyrate while suppressing pathogenic strains such as *Odoribacter* and LPS-producing *Marinifilaceae*, thereby mitigating inflammatory responses and CRC progression (Yan et al., 2022).

Rare ginsenoside Rh4 (Figure 2, **5**), a crucial active component in ginseng, has been stated to possess the capability of inducing apoptosis in tumor cells and ameliorating antibiotic-induced intestinal dysbiosis and inflammation (Wu et al., 2018; Bai et al., 2024) discovered that 7α-hydroxy-steroid dehydrogenas activity and UDCA production were enhanced by rare ginsenoside Rh4 by enriching *Akkermansia muciniphila*, a bile acid metabolism probiotic, in AOM/DSS-induced mouse model, which subsequently activated the farnesoid X receptor while modulating the TLR4/NF-κB inflammatory cascade, effectively restoring intestinal barrier integrity, suppressing colonic inflammatory processes and impeding CRC progression.

New Houttuynia sodium folate (Figure 2, **6**), originating from the herb Houttuynia cordata (Jia et al., 2022), exhibits potent antimicrobial effects against *F. nucleatum* (Jia et al., 2022). found that new Houttuynia sodium folate reduced *F. nucleatum* load in tumor tissue and suppressed tumor progression in *F. nucleatum* mouse xenografts by targeting membrane-associated FadA and modulation of F. *nucleatum*-associated inflammatory pathways, while the damaged intestinal barrier was also improved.

*Panax notoginseng* saponin, recognized as the primary bioactive constituent derived from *P. notoginseng* extracts, comprises ginsenoside Rb1, Rg1 and notoginsenoside R1 as its principal components (Zhang H. et al., 2024). Colon tumorigenesis and development was alleviated by *P. notoginseng* saponin via restoring the richness and diversity of intestinal flora, in particularly, by increasing the abundance of *Akkermansia spp*, which is inversely associated with CRC progression. Furthermore, gut microbiota biotransformation analysis revealed ginsenoside compound K (Figure 2, 3) as the principal microbial metabolite of *P. notoginseng* saponin, exhibiting potent antiproliferative effects against human CRC cell lines through distinct molecular mechanisms (Chen et al., 2020).

Pien Tze Huang, a well-established traditional medicinal formulation, has been proven to prevent and treat CRC through microbial-dependent and non-microbial-dependent mechanisms (Chen et al., 2022). As a microbial-dependent mechanism, Pien Tze Huang enhances intestinal barrier integrity by modulating gut microbial composition, notably elevating beneficial strains like *Pseudobutyrivibrio xylanivorans* and *Eubacterium limosum* while suppressing pathogenic species including *Aeromonas veronii*, *Campylobacter jejuni*, *Collinsella aerofaciens*, and *Peptoniphilus harei*. Concurrently, this herbal preparation elevates protective metabolites such as taurine derivatives, bile acids, and unsaturated fatty acids. Through non-microbial pathways, PZH downregulates oncogenic signaling cascades including PI3K-Akt activation and pro-inflammatory mediators like IL-17, TNF, and chemokine networks. Experimental evidence reveals that specific bioactive constituents [ginsenoside-F2 (Figure 2, **7**) and ginsenoside-Re (Figure 2, **8**)] exhibit anti-proliferative effects against CRC cell lines and patient-derived organoids, with additional efficacy observed in AOM/DSS-induced CRC models (Gou et al., 2023).

Curcumin (Figure 2, **9**), a primary polyphenolic compound derived from turmeric rhizomes (Anas et al., 2024). Curcumin treatment has been shown can effectively reverses microbial dysbiosis in CRC mice by restoring core microbiota diversity and abundance, this treatment suppresses pathogenic genera including *Ileibacterium*, *Monoglobus* and *Desulfovibrio* while enhancing beneficial bacterial populations such as *Clostridia_UCG-014*, *Bifidobacterium* and *Lactobacillus* (Deng et al., 2024). 13 different metabolites were identified, and curcumin reduced levels of ethylsuximine, xanthine, and 17-β-estradiol 3-sulfate-17 - (β-D-glucuronide), which were augmented in the CRC model group. In contrast, glutamylleucine, γ-glutamylleucine, liquiritin, ubenimex, 5'-deoxy-5 ′-fluoruridine, 7, 8-dihydroterophenic acid, ribenzapril, heterosin A, and 7,4'-dihydroxy-8-methylflavane were reduced in the CRC group, but were significantly upregulated by curcumin. However, the therapeutic mechanisms and clinical relevance of these biochemical alterations in CRC pathogenesis demand more comprehensive exploration.

## 3 Natural derived small molecule compounds treat colorectal cancer by promoting ferroptosis

Ferroptosis is a recently identified type of regulated cell death which is triggered through iron-mediated lipid peroxidation processes, showing therapeutic potential for CRC management (Li et al., 2020). This cell death pathway primarily involves catalytic oxidation of membrane-bound polyunsaturated fatty acids by ferrous iron or lipoxygenases, generating lipid peroxides that culminate in cellular demise (Dixon et al., 2024). As a crucial component in ferroptosis regulation, glutathione (GSH) plays dual roles in supporting immune homeostasis and combating oxidative stress. Serving as the primary cofactor for GPX4 (glutathione peroxidase 4), this tripeptide enables the enzyme’s lipid repair capabilities by reducing oxidized membrane components (Weaver and Skouta, 2022). Depletion of GSH reserves incapacitates GPX4’s redox-regulatory functions, precipitating lethal lipid peroxidation in CRC cells (Niu et al., 2021). The cystine/glutamate antiporter (System XC-), functioning as the principal antioxidant pathway in mammalian cells, has emerged as a critical ferroptosis modulator through its cystine transport activity (Parker et al., 2021). The reverse transport function of xCT regulates the synthesis of GSH by exchanging glutamate for cysteine, thereby helping to balance excessive hydrogen peroxide (Lim et al., 2019). Within ferroptosis mechanisms, solute carrier family 7 member 11 (SLC7A11) serves as a crucial regulatory component (Liu Z. et al., 2022). Functioning as a ferroptosis suppressor, this protein operates within the System XC- complex by controlling cellular cystine absorption (Lee and Roh, 2022). Disruption of the XC- system diminishes cystine availability, subsequently halting GSH biosynthesis and initiating iron-dependent cell death pathways (Sun K. et al., 2023). There are many reports NDSMs treat CRC by promoting ferroptosis (Figure 3).

Resveratrol (Figure 2, **10**), a plant-derived non-flavonoid polyphenol, functions as a phytoalexin with demonstrated therapeutic potential in cancer treatment (Vikal et al., 2024). This bioactive compound, naturally present in wine and grape products, exhibits multifaceted biological activities including oxidative stress reduction, inflammation control, and cardioprotective effects. Resveratrol potentiates ferroptosis through dual inhibition of SLC7A11 and GPX4 expression, thereby amplifying reactive oxygen species (ROS) generation and lipid peroxidation in CRC cells (Zhang Z. et al., 2022).

Angelic acid (Figure 2, **11**), a bioactive constituent derived from the rhizomes of *Angelica sinensis (Oliv.)* Diels, has been identified as a ferroptosis inducer in CRC cell (Wen et al., 2025; Cao et al., 2024) found angelic acid destabilizes nuclear factor erythroid 2-related factor 2 (NRF2), a crucial regulator of oxidative stress responses, through protein degradation mechanisms, as evidenced including elevated malondialdehyde (MDA) levels, enhanced lipid peroxidation, and increased expression of CHAC1 and PTGS2 -effects that were reversed upon administration of the ferroptosis inhibitor Fer-1. Mechanistically, no significant changes in the mRNA levels of GPX4 and NRF2 were detected after angelic acid treatment, but the expression of NRF2 was weakened in a concentration-dependent manner. The cellular thermal shift assay and cycloheximide chase assay demonstrated that that angelic acid affected NRF2 stability by increasing the K48-ubiquitination level of NRF2. The reversal of NRF2-mediated suppression on CRC cell viability through genetic overexpression further confirms that angelic acid physically interacts with NRF2 to accelerate its proteasomal breakdown, consequently initiating ferroptotic cell death mechanisms in CRC cells. Additionally, the syngeneic mouse model revealed that the sensitivity of CRC cells to ferroptosis inducers, sulfasalazine, was enhanced by angelic acid and no toxicity *in vivo* showed, basically, demonstrating the promising feasibility of angelic acid as a dietary therapeutic agent for CRC treatment.

Ferroptosis represents an iron-dependent oxidative cell death mechanism driven by Fenton reaction-generated reactive Oxygen Species (ROS) (Zhang X. et al., 2024). Malignant cells exhibit heightened iron demands to support their rapid proliferation, making them particularly vulnerable to iron-mediated cytotoxicity (Li et al., 2025). During iron deficiency, cells release ferritin, their primary iron storage complex, through regulated pathways. Iron storage protein, ferritin, is usually released by the cell when it urgently needs iron. Nuclear receptor coactivator 4 (NCOA4) is an important transcriptional regulatory factor (Yin et al., 2024). The NCOA4-mediated degradation pathway, termed ferritinophagy, involves selective recognition of ferritin by autophagic vesicles for lysosomal breakdown (Santana-Codina et al., 2021). This process elevates intracellular iron concentrations, exacerbating oxidative damage through lipid peroxidation cascades that initiate ferroptotic cell death (Hoelzgen et al., 2024).

Emodin (Figure 2, **12**), a bioactive compound exhibiting significant anticancer, hepatoprotective, anti-inflammatory and antimicrobial properties, is widely present in various medicinal herbs including *Rheum palmatum*, and *Polygonum multiflorum* (Hassan et al., 2024; Shen et al., 2024) demonstrated that this phytochemical triggers ferroptosis in CRC cells through dual mechanisms involving NCOA4-regulated ferritinophagy and suppression of the NF-κB signaling pathway. After emodin treatment, ROS generation, lipid peroxidation levels, MDA levels, iron levels, and the expressions of NCOA4 and transferrin receptor (TFRC) increased in CRC, while GSH/GSSG ratio, System Xc-, GPX4 and ferritin heavy chain 1 (FTH1) expression decreased. Crucially, genetic ablation of NCOA4 reversed emodin-induced iron overload, confirming ferritinophagy’s essential role in mediating iron accumulation during this process. The study further established a mechanistic link between NF-κB pathway inhibition and ferroptosis induction, with emodin treatment significantly downregulating components of this inflammatory signaling cascade. This indicates that coordinated regulation of ferroptosis and inflammatory pathways underlies emodin’s therapeutic potential against CRC. Experimental data revealed downregulation of NF-κB and IKK signaling pathway components, indicating emodin-mediated suppression of NF-κB activation. Pharmacological activation of NF-κB using PMA significantly counteracted emodin’s effects on critical regulators including antioxidant proteins (SLC7A11, GPX4), autophagy markers (LC3B, P62), and iron metabolism proteins (NCOA4, FTH1) in CRC cells and PMA partially reversed cytoplasmic iron accumulation, as well as MDA and lipid peroxidation. These findings collectively demonstrate that NF-κB pathway inhibition plays a pivotal role in mediating emodin-triggered ferroptosis in CRC through modulation of iron homeostasis and oxidative stress mechanisms.

Ginsenoside Rh3 (Figure 2, **13**), a bioactive triterpenoid compound derived from *Panax ginseng* C. A. Mey roots, demonstrates significant anticancer properties (Xu et al., 2023; Wu et al., 2023a) revealed ginsenoside Rh3 can effectively eliminate CRC cells through both pyroptosis and ferroptosis. Pyroptosis involves Caspase-1-mediated activation of Gasdermin proteins, particularly Gasdermin D, which manifests through cellular membrane perforation and subsequent release of inflammatory mediators into the extracellular environment (Shi et al., 2017). The STAT protein family, particularly STAT3, serves as critical regulators of cellular signaling networks, coordinating essential biological functions ranging from cell cycle progression to angiogenesis. Under physiological conditions, STAT3 undergoes transient phosphorylation to mediate cytokine-induced transcriptional responses, maintaining strict regulation of cellular processes (Kim et al., 2018). Ginsenoside Rh3 modulates oncogenic signaling pathways by interfering with nuclear translocation of redox regulators. While STAT3 demonstrates constitutive activation across numerous malignancies, pharmacological intervention with ginsenoside Rh3 specifically attenuated tyrosine-phosphorylated Stat3 levels while elevating total p53 tumor suppressor expression. Although whole-cell analyses showed minimal alterations in total Stat3 and NRF2 concentrations, nuclear fractionation revealed substantial suppression of NRF2 nuclear accumulation following ginsenoside Rh3 exposure. This impaired nuclear localization consequently diminished HO-1 transcriptional activity, thereby enhancing NLRP3 inflammasome assembly and caspase-1 activation. The activated caspase-1 protease cleaves Gasdermin D to execute pyroptotic cell death. Concurrently, ginsenoside Rh3-mediated nuclear exclusion of NRF2 disrupts xCT/SLC7A11-dependent glutathione synthesis, precipitating iron overload, lipid peroxidation (evidenced by MDA accumulation), and catastrophic GSH depletion - hallmark events culminating in ferroptotic cell death (Wu et al., 2023a).

## 4 Natural derived small molecule compounds treat colorectal cancer by promoting apoptosis

Apoptosis is defined by distinct cellular changes including nuclear condensation, chromatin fragmentation, membrane blebbing, and reduced MMP activity (Bertheloot et al., 2021). The mitochondrial apoptotic pathway is primarily regulated through interactions between Bcl-2 family members and caspase activation (Czabotar and Garcia-Saez, 2023). As opposing regulators within this protein family, Bcl-2 demonstrates anti-apoptotic properties by maintaining mitochondrial membrane integrity and preventing cytochrome C release (Czabotar and Garcia-Saez, 2023). Conversely, the pro-apoptotic Bax protein promotes mitochondrial outer membrane permeabilization, facilitating cytoplasmic release of cytochrome C and apoptotic activators to initiate cell death cascades (Wolf et al., 2022). Bax increases apoptosis by binding to Bcl-2, disrupting the protective effect of Bcl-2 on apoptosis (Edlich, 2018) Concurrently, dysregulated cell cycle progression contributes to malignant proliferation, with cyclin D1 overexpression being frequently observed in cancerous growth patterns (Wang et al., 2023b). Many NDSMCs have been shown to exert therapeutic effects through multifaceted pathways, including modulation of Bcl-2 protein clusters, Caspase enzyme systems, and cyclin D1 expression. These biochemical interventions demonstrate capacity to control mitotic progression, activate programmed cell death pathways, and suppress CRC.

### 4.1 Natural derived small molecules induce apoptosis of colorectal cancer cells by inhibiting nuclear factor kappa-beta (NF-κB) signaling pathway

The NF-κB signaling cascade serves as a central modulator in numerous biological functions including immune regulation, inflammatory responses, and cellular homeostasis (Mao et al., 2025). This molecular pathway exhibits responsiveness to diverse stimuli encompassing inflammatory mediators, oxidative stressors, radiation exposure, and microbial pathogens, subsequently governing transcriptional activation of multiple genetic targets (Guo et al., 2024).

NF-κB represents an umbrella term for dimeric transcription factor complexes predominantly composed of structural subunits (Yu et al., 2020). In unactivated state, NF-κB is located in the cytoplasm and binds to the inhibitory protein IκB, thus remaining in an inactive state (Mitchell and Carmody, 2018). Cellular activation triggered by specific stimuli induces IκB phosphorylation followed by proteasomal degradation, thereby liberating NF-κB complexes (Guo et al., 2024). The liberated dimers subsequently translocate to the nuclear compartment where they initiate the transcription of pro-inflammatory cytokines and survival factors, processes critically involved in inflammatory pathogenesis and oncogenic development (Guo et al., 2024).

Asiaticoside (Figure 2, **14**), the primary bioactive component derived from *Centella asiatica* (Umbelliferae family), demonstrates notable anti-inflammatory properties as documented in prior research (Bandopadhyay et al., 2023). I-κBα phosphorylation was found diminished by Asiaticoside in human CRC cell lines (HCT116, SW480, LoVo) concentration-dependently, which effectively blocks the nuclear translocation of the P65 subunit, indicating its potential to suppress colorectal tumor progression and stimulate apoptotic mechanisms through NF-κB pathway inhibition. Concurrently, researchers observed marked downregulation of CDK4 and Cyclin D1 expression alongside enhanced caspase-9 and caspase-3 activation. These cellular changes, coupled with a reduced Bcl-2/Bax mRNA ratio, collectively contribute to G0/G1 phase cell cycle arrest and programmed cell death induction (Figure 3) (Zhou et al., 2020).

The TNF-activated NF-κB signaling pathway serves as a critical mediator in cancer pathogenesis and represents a promising therapeutic target for pharmacological intervention (Zhang T. et al., 2021). Roburic acid (Figure 2, **15**), a recently identified tetracyclic triterpene compound extracted from *oak galls*, exhibits notable anti-inflammatory properties (Wang L. et al., 2024; Xu et al., 2022) reveals roburic acid’s capacity to suppress IKKα/β phosphorylation, inhibit IκBα degradation, and prevent p65 nuclear translocation in TNF-stimulated systems. This molecular mechanism was observed in both *in vitro* models using CRC cells and *in vivo* studies with xenografted nude mice, demonstrating roburic acid’s ability to downregulate NF-κB-regulated survival proteins such as XIAP, Mcl-1, and Survivin. These findings collectively establish RA roburic acid’s anti-proliferative effects on CRC cells through NF-κB pathway inhibition roburic acid was found inhibited the phosphorylation of IKKα/β, IκBα and p65, degradation of IκBα, nuclear translocation of p65 and expression of NF-κB target gene, including that of XIAP, Mcl-1, and Survivin, in TNF-induced CRC cells and xenografted nude mice, indicated that inhibited the growth of human CRC cells was inhibited by roburic acid via inhibiting NF-κB signaling pathway (Figure 4) (Xu et al., 2022).

### 4.2 Natural derived small molecules induce apoptosis of colorectal cancer cells by inhibiting PI3K/Akt signaling pathway

The Phosphatidylinositol 3-kinase/protein kinase-B (PI3K/Akt) cascade represents a crucial intracellular signaling mechanism activated by numerous oncogenic factors and receptor tyrosine kinases (Thapa et al., 2020). This pathway’s core components include PI3K and the serine-threonine kinase Akt, with the latter serving as PI3K’s primary downstream effector that directly mediates its activation signals (Khezri et al., 2022). Research across various malignancies has demonstrated that dysregulation of this signaling axis drives oncogenic processes through sustained activation, facilitating tumor cell proliferation, metabolic adaptation, and survival signaling networks (Khezri et al., 2022).

Periplocymarin (Figure 2, **16**), a cardiac glycoside derived from *Periploca sepium*, exhibits potential anticancer properties (Cheng Y. et al., 2021; Cheng et al., 2021b) demonstrated that this compound exerts therapeutic effects against CRC through impairment of PI3K/AKT pathway functionality, which mechanism involves triggering apoptotic processes and inducing cell cycle arrest at the G0/G1 phase, achieved through suppression of IRS1 expression and phosphorylation of PI3K/AKT proteins. Additionally, periplocymarin modulates the expression levels of Bcl-2 family members, survivin, p21, and cyclin D1 proteins, which collectively contribute to its anticancer efficacy.

Dihydroartemisinin (Figure 2, **17**), a derivative of artemisinin, demonstrates potent antitumor effects through inducing programmed cell death, suppressing neoplastic growth, and impeding cellular proliferation and motility (Olatunde et al., 2023; Chen and Yao, 2023) found that dihydroartemisinin effectively curtailed CRC RKO cell proliferation and migration while stimulating apoptosis through G2/M phase cell cycle arrest, which mechanism involves suppression of PI3K/AKT signaling pathway activation, evidenced by reduced phosphorylation of p38 MAPK, PI3K, and AKT proteins. The treatment concurrently downregulated AKT expression while elevating MMP-9 protein levels and Caspase-3/9 mRNA concentrations. Notably, dihydroartemisinin administration increased cleaved Caspase-9/Caspase-9 ratios and elevated Bax/Bcl-2 protein expression proportions. In animal models, dihydroartemisinin outperformed cisplatin by enhancing body mass and substantially decreasing serum TNF-α concentrations, tumor mass, and volumetric dimensions with 41.45% tumor growth inhibition. Parallel *in vitro* experiments confirmed dihydroartemisinin capacity to suppress malignant cell proliferation/migration, induce apoptotic processes, and mediate G2/M phase cycle arrest in cultured cells.

Dieckol (Figure 2, **18**) is a polyphenol extracted from brown algae *Ecklonia cava* (Rajan et al., 2021). Dieckol successfully inhibited the PI3K/AKT/mTOR signaling pathway by significantly reducing the expression and protein levels of Cyclin-D1, PCNA, Bcl-2, p-P13K, AKT, mTOR and other apoptosis-related genes, thereby inducing the apoptosis of HCT-116 cells (Dai et al., 2023). Notably, dieckol also significantly increased the concentration of ROS in HCT-116 cells, which are molecules with unpaired valence shell electrons, also known as free radicals. ROS are incredibly active and inflict a large amount of oxidative damage to the organism, which is considered as the main factor of apoptosis.

### 4.3 Natural derived small molecules induce apoptosis of colorectal cancer cells by inhibiting hedgehog signaling pathway

Hedgehog (HH) signaling pathway is a classical signaling pathway that controls embryonic development and maintains crucial functions in embryonic development and cell growth and proliferation after embryo formation (Martelli et al., 2023).

The HH signaling pathway consists of HH ligands (SHH, DHH, IHH), 2 cell membrane receptors Patched (Ptc) and Smoothened (Smo), and Glioma 2/3 (Glioma-associated oncogene) homolog, serine/threonine protein kinase Fused (Fu), negative regulatory protein Suppressor of Fused (SuFu), microtubule associated protein Kinesin family member 7(KIF7), protein kinase A (PKA), etc. (Jing et al., 2023). HH receptor patch 1 (PTCH1) and HH receptor patch 2 (PTCH2) encode the negative regulatory proteins Patched1 and Patched2 in the Hedgehog signaling pathway, respectively (Zhong and Wang, 2022). Smo encodes the positive regulatory protein Smoothened in the HH signaling pathway. The transcription factors Gli (1, 2 and 3) are the main downstream executive factors of HH activation and key final outputs of HH (Lou et al., 2020). Gli1 is an HH response gene product, which only acts as a transcriptional activator and participates in the formation of positive feedback loops during pathway activation (Sigafoos et al., 2021). Gli2 and Gli3 are the major transcriptional activators and suppressors, respectively (Yoshida and Yoshida, 2025). Under ligand-deficient conditions, PTCH1 actively suppresses Smo activity by blocking its translocation into primary cilia (Cong et al., 2025). When HH ligand binds to Ptc, this mutual inhibition is relieved and Smo signal is excite (Jing et al., 2023). Ligand-receptor interaction between HH and Ptc disrupts this mutual antagonism, triggering Smo signal transduction (Li et al., 2018). Aberrant activation of this signaling cascade is frequently observed in multiple malignancies. Emerging evidence confirms the Hedgehog pathway’s regulatory involvement in CRC, particularly through SHH-mediated stimulation of neovascularization, cellular multiplication, and metastatic progression, while IHH downregulation emerges as a precursor event in CRC pathogenesis (Wang et al., 2023a).

Sun et al. (2023b) demonstrated that BBR (Figure 2, **4**) effectively suppresses the HH signaling pathway in both *in vitro* models and HCT116 xenograft tumors. This inhibition manifests through downregulation of SHH, Ptch1, SMO, Gli1, and c-Myc expression while elevating SUFU levels in CRC cells. Furthermore, experimental evidence reveals BBR’s capacity to trigger programmed cell death and arrest the cell cycle at the G0/G1 phase. The compound concurrently diminishes G2/M and S phase distribution through modulation of Bcl-2 and Bax expression levels, accompanied by reduced mitochondrial membrane potential and decreased cyclin D1 production in CRC cells.

Baicali (Figure 2, **19**), a naturally occurring flavonoid compound extracted from the medicinal herb *Scutellariabaicalensis Georgi* (Ibrahim et al., 2022), demonstrates regulatory effects on inflammatory cytokines and Hedgehog pathway components. Experimental studies reveal BC downregulates mRNA and protein expression of IL-1β, IL-6, TNF-α, SHH, SMO, and Gli1 while upregulating SUFU expression in SW620 CRC cells. In CRC models, BC administration significantly reduces tissue levels of pro-inflammatory cytokines (IL-1β, IL-6, TNF-α) and modulates Hedgehog signaling markers - suppressing SHH, SMO, and Gli1 protein expression while enhancing SUFU protein production. These findings suggest baicali anti-proliferative and pro-apoptotic effects on CRC cells may operate through Hedgehog pathway inhibition (Lin, 2023).

Scutellarin (Figure 2, **20**) is the another active ingredient of *Scutellariabaicalensis Georgi* (Liao et al., 2021). Scutellarin inhibited the activity of the HH signaling pathway in CRC tissues and SW480 cells, reducing the expression of SHH, Ptch1, Smo, and Gli1, while increasing the level of SUFU. At the same time, scutellarin treatment could inhibit the phosphorylation and nuclear translocation of NF-κB p65 in response to TNF-α stimulation in IEC-6 cells to exert anti-inflammatory effects. The literature confirms that NF-κB can induce the expression of SHH and IL-1β and IL-6 by activating the HH signaling pathway. Activated NF-κB can induce overexpression of SHH, activating the HH signaling pathway. Therefore, the blockade of NF-κB signaling may inhibit the expression of SHH and thus the HH signaling axis (Zeng et al., 2022). Future research directions should prioritize mechanistic studies exploring SL’s regulatory effects on the HH-NF-κB crosstalk in CRC.

### 4.4 Natural derived small molecules induce apoptosis of colorectal cancer cells by inhibiting Wnt/β-catenin signaling pathway

In the majority of CRC cases, upregulated expression of key genes within the Wnt/β-catenin pathway contributes to cell cycle disruption in malignant cells, promoting accelerated invasion and metastatic progression (Liu J. et al., 2022). The nuclear accumulation of β-catenin directly correlates with pathway activation intensity, making its intracellular concentration regulation fundamental for controlling this signaling cascade (Liu J. et al., 2022). Under normal physiological conditions without Wnt stimulation, cytoplasmic β-catenin undergoes phosphorylation through the APC/Axin/GSK3β destruction complex, subsequently targeted for proteasomal degradation to maintain subthreshold concentrations (Maurice and Angers, 2025). When Wnt signaling is triggered by ligand binding to membrane receptors, GSK3β undergoes phosphorylation-induced functional impairment, enabling β-catenin stabilization and cytoplasmic accumulation (Shah and Kazi, 2022). This stabilized signaling molecule subsequently transports into nuclear compartments, initiating transcriptional activation of oncogenic targets including CD133, CD44, ALDH isoforms, c-Myc proto-oncogenes, and cyclin-dependent kinase regulators (Chen et al., 2012). Numerous studies have demonstrated that constitutive activation of this pathway represents a central driver in CRC.

Ursolic acid (Figure 2, **21**), a naturally occurring pentacyclic triterpene compound, is widely distributed in numerous edible plants, herbal medicines, and culinary spices (Arulnangai et al., 2025). Ursolic acid was found greatly inhibited the proliferation, migration, and clonality of SW620 cells; induced apoptosis; and arrest the cell cycle in the G0/G1 phase, accompanied by decreased activity of the Wnt/β-catenin signaling pathway. Furthermore, *in vivo* investigations revealed ursolic acid administration markedly inhibited tumor progression in xenograft models, ameliorated histopathological characteristics, enhanced programmed cell death, and induced cell cycle blockade in CRC tissues through downregulation of Wnt/β-catenin signaling components (Zhao et al., 2023).

Apigenin (Figure 2, **22**), a flavonoid, is abundantly present in various fruits and vegetables from tropical regions, with celery being a particularly rich source (Yan et al., 2014). Zhao et al. (2023) reveals that apigenin counteracts LiCl-induced activation of β-catenin/T-cell factor/lymphoid enhancer-binding factor signaling cascades, showing concentration-dependent efficacy against this Wnt pathway activator. This action prevents nuclear translocation of β-catenin, consequently blocking the transcriptional activation of Wnt-regulated target genes.

Lonchocarpin (Figure 2, **23**) was initially extracted from *Lonchocarpus sericeus* (synonymously termed Derris sericeus) through the pioneering work of Baudrenghien’s research group in 1949 (do Nascimento and Mors, 1972). Subsequent investigations by Predes et al. (2019) identified lonchocarpin as a novel Wnt/β-catenin pathway inhibitor that disrupts β-catenin nuclear translocation, thereby decreasing its nuclear accumulation. This compound additionally demonstrated inhibitory effects on the constitutively active TCF4 variant dnTCF4-VP16. *In vivo*, *Xenopus laevis* embryology assays showed that lonchocarpin acts at the transcriptional level. Embryological studies using *X. laevis* models revealed lonchocarpin’s transcriptional-level activity. Experimental evaluations using colorectal carcinoma cell lines (HCT116, SW480, DLD-1) demonstrated lonchocarpin’s capacity to suppress both cellular migration and proliferation, while showing no cytotoxic effects on the non-malignant intestinal IEC-6 cell line. Furthermore, *in vivo* testing using AOM/DSS-induced murine CRC models confirmed lonchocarpin’s tumor-suppressive properties. Notably, multiple solid malignancies including colorectal carcinomas contain cancer stem cells-a distinct cellular subgroup demonstrating self-renewal capacity and multilineage differentiation potential. These neoplastic stem cells significantly contribute to tumor maintenance and progression through their unique biological properties.

The initiation, progression, metastatic spread, chemoresistance, and relapse of malignancies are closely associated with CRC stem cells (CRCSCs), making these cells a promising therapeutic target for impeding metastasis and recurrence in CRC (Chu et al., 2024). As the primary bioactive compound in green tea, (−)-Epigallocatechin-3-Gallate (EGCG) (Figure 2, **24**) has been shown to suppress CRCSCs proliferation and promote apoptotic cell death through modulation of the Wnt/β-catenin signaling cascade (Chen et al., 2017). The expression levels of CD133, CD44, ALDHA1, which are CRC markers in CRC, as well as the protein and mRNA levels of cell cycle protein D1 and PCNA were observed to be downregulated with EGCG treatment. At the same time, EGCG treatment led to a downregulation of Bcl-2 expression and an upregulation of Bax, caspases (3, 8, and 9) levels.

### 4.5 Natural derived small molecules induce apoptosis of colorectal cancer cells by inhibiting MAPK signaling pathway

Extensive research has demonstrated that Mitogen-activated protein kinase (MAPK) serves as a central mediator in translating external signals into diverse biological responses such as cellular development, motility, multiplication, specialization, and programmed cell death (Braicu et al., 2019). The mammalian MAPK family includes ERK, JNK and P38, etc. (Wu et al., 2023b). Gadd45a protein, characterized by its acidic nature and spherical conformation, belongs to a conserved group of molecules associated with growth suppression and genomic instability (Rostami et al., 2025). This multifunctional regulator participates in critical cellular operations encompassing genetic material restoration, division cycle control, cell death mechanisms, neoplastic transformation, and blood vessel formation. Research indicates that Gadd45a’s apoptosis-promoting effects frequently operate through activation pathways involving both p38 and JNK kinases (Yue and López, 2020).

Bavachin (Figure 2, **25**), a dihydroflavonoid derived from Psoralea species, exhibits diverse pharmacological propertie (Wang et al., 2025). It was found that bavachin’s capacity to stimulate phosphorylation of p38, JNK, and ERK kinases in both HCT 116/HT-29 cell lines and murine xenograft models. This biochemical activation coincided with upregulated Gadd45a expression, consequently triggering MAPK pathway activation that suppresses CRC cell proliferation while promoting apoptotic mechanisms (Wang M. et al., 2023).

HIF-1 is a transcription factor that regulates the expression of target genes related to oxygen homeostasis under hypoxic conditions, thereby promoting tumor development and progression,and he overexpression of HIF-1α is closely associated with poor prognosis in cancer patients (Pandey et al., 2025). Kaempferol (Figure 2, **26**) is a naturally occurring flavonoid compound found in various fruits and vegetables, and it has attracted attention due to its potential anti-cancer effects. Mechanistic studies have shown that kaempferol can dual regulate the transcriptional activity of HIF-1α and MAPK signaling (p-ERK/p-38), as well as ROS-induced DNA damage and intrinsic cell apoptosis (cleaved caspase-3/9 and Bcl-2 protein expression), effecting on angiogenesis, EMT, and survival pathways significantly reduce the proliferation, invasion, and metastasis abilities of hypoxic colon cancer cells, indicating that kaempferol can serve as an innovative multi-pathway inhibitor (Haroon and Kang, 2025).

## 5 Natural derived small molecule compounds treat colorectal cancer by promoting autophagy

Autophagy is a degradative metabolic pathway, mainly relies on lysosomes to clear damaged or aged cellular organelle (Liu S. et al., 2023). Among autophagy-regulating proteins, ATG7 represents a crucial molecular player that primarily facilitates autophagosome biogenesis through its enzymatic functions (Liu et al., 2024).

Celastrol (Figure 2, **27**) is a bioactive component extracted from *Tripterygium wilfordii*, and has shown may induce autophagy in CRC by targeting Nur77 which is a pro-cancer regulator and upregulating ATG7 (Zhang W. et al., 2022), which offers novel perspectives on celastrol’s antitumor potential in CRC. Celastrol administration effectively suppressed tumor progression in mice bearing CRC xenografts, potentially mediated through Nur77 downregulation-induced ATG7 upregulation. Notably, celastrol-treated human CRC cell lines (HCT-116 and SW480) exhibited diminished clonogenic capacity alongside elevated pro-apoptotic Bax and cleaved PARP levels, coupled with reduced anti-apoptotic Bcl-2 expression. Concurrently, autophagy markers showed significant alterations: decreased p62 protein levels contrasted with increased Beclin-1 expression, while crucially impairing the Bcl-1/Bcl-2 interaction required for autophagy initiation. Additionally, celastrol promoted the conversion of LC3-I to lipidated LC3-II in CRC cells, which is essential for the formation of autophagosomes.

The mammalian target of rapamycin (mTOR), an atypical serine/threonine kinase, serves as a pivotal regulator of cellular processes including proliferation, programmed cell death, autophagy, and metabolic regulation (Panwar et al., 2023). Aberrant activation of this signaling molecule stimulates cellular proliferation and metastatic potential, establishing its central role in pathway modulation (Holroyd and Michie, 2018). Dysregulation of the PI3K/Akt/mTOR axis frequently occurs in various malignancies. Rhein (Figure 2, **28**), a bioactive anthraquinone compound derived from rhubarb species (Cheng L. et al., 2021) demonstrates significant antitumor effects through mTOR interaction. Zhang et al. (2021b) revealed that rhein administration effectively suppresses CRC cell proliferation and metastatic behavior through direct mTOR binding and subsequent pathway inhibition. Mechanistically, this natural compound induces G1 phase arrest by modulating cell cycle regulators including cyclin A1, E1, D1, and CDK2 expression. Concurrent elevation of apoptotic mediators (p53, phosphorylated p53, activated caspase 3 and Bax) confirms rhein’s pro-apoptotic capacity in malignant colon cells. The critical role of epithelial-mesenchymal transition processes in tumor progression further underscores Rhein suppressed CRC cell motility and metastatic potential through modulation of epithelial-mesenchymal transition markers, including increased E-cadherin production and decreased expression of N-cadherin and vimentin. The influence of rhein on the mTOR signal transduction pathway in CRC cells is reflected in the fact that it can directly bind to mTOR and downregulate mTOR expression. mTOR is crucial for the activation of HSF1 and the synthesis of HSP90, and the activation of HSF1 and the synthesis of HSP90 in many cancers are significantly associated with tumor metastasis and death. The protein levels of HSF1 and HSP90 in CRC cells were downregulated by RE treatment. Furthermore, the ubiquitin-proteasome pathway plays an important role in protein degradation, and RE can promote the degradation of mTOR via the ubiquitin-proteasome. Similarly, RE showed significant tumor growth inhibition in xenografted mouse models without significant toxicity. RE is, therefore, a potent anticancer agent that may help prevent and treat CRC.

Myriceti (Figure 2, **29**), a flavonoid pigment abundant in fruits, herbs, and nuts (Zhu et al., 2020). Studies (Zhu et al., 2020) reveals that myriceti modulates programmed cell death mechanisms through PI3K/Akt/mTOR pathway inhibition. Myriceti diminishes Bcl-2/Bax protein ratios while promoting apoptosis in HCT116 and SW620 cell lines. Morphological changes observed under optical microscopy after 48-h exposure to 50–100 μmol/L myriceti included cellular rounding, membrane blebbing, and cytoplasmic autophagic vacuoles. The autophagy process was evidenced by LC3-I to LC3-II transformation, with immunoblotting showing increased LC3-II/β-actin ratios. Concurrent elevation of Beclin-1/β-actin expression, indicative of autophagosome formation, was observed alongside these molecular changes. Exposure to myriceti triggered a dose-responsive escalation in autophagic activity within HCT116 and SW620 cell lines, with microscopic analysis revealing enhanced autophagic vesicle formation compared to control groups. Phospho-PI3K, phospho-Akt, and phospho-mTOR levels showed dose-dependent reductions in both cell models, while total protein levels of these signaling molecules remained statistically comparable across treatment conditions. Notably, the phosphorylation ratios of Akt and mTOR relative to their total forms were markedly diminished. These collective findings indicate that myriceti administration activates autophagic processes in CRC cells through modulation of the PI3K/Akt/mTOR signaling axis.

The Aurora kinase family, comprising three subtypes (Aurora A, B, and C), consists of serine/threonine kinases essential for regulating mitotic progression (Kolli, 2025). Elevated Aurora gene expression through amplification and increased mRNA/protein production has been documented across multiple malignancies including prostate, breast, pancreatic, and ovarian cancers. Aurora A demonstrates specific localization at mitotic spindle poles and centrosomes (Lin et al., 2020), positioning it as a promising therapeutic target in oncology due to its functional interaction with p53 through phosphorylation-mediated degradation (Magnaghi-Jaulin et al., 2019). Derived from the traditional Chinese medicinal plant *Salvia miltiorrhiza Bunge* (Danshen), Tanshinone (Figure 2, **30**) represents a bioactive constituent (Naz et al., 2020) with demonstrated anticancer properties. Studies (Lu et al., 2016) have shown that the mechanism by which Aurora A-p53 axis may inhibit the growth of CRC cells may differ depending on the intrinsic characteristics of the tumor cells. In HCT116 cancer cells, after 24 h of treatment, tanshinone significantly increased the proportion of G2/M and 4N cells, decreased the proportion of S and G0/G1 cells, and reduced the expression levels of CDK4, cyclin D1, c-PARP, Bax, and Bcl-2 proteins, indicating cell cycle arrest and increased cell apoptosis.

## 6 Progress in clinical research of natural derived small molecules

The finally goal of basic research on NDSMCs is the successful application of safe and effective drugs to clinical patients. Through clinical research network station (https://clinicaltrials.gov/) to retrieve the data about the NDSMCs against CRC clinical research data (Table 2). Lots and quality NDSMCs have been studied against CRC, which undoubtedly confirms the reproducibility and translational potential of their anti-CRC effects.

**TABLE 2:** Clinical trial of colorectal cancer with natural derived small molecule compounds.

Project title	Compound	Status	Registration no.
Phase 2 Study with Abraxane (Nab®Paclitaxel) in Metastatic Colorectal Cancer	Paclitaxel	Phase 2	NCT02103062
Curcumin Biomarkers	Curcumin	Phase 1	NCT01333917
Clinical Study Evaluating the Anticancer Effect of Pentoxiphylline in Patients with Metastatic Colorectal Cancer (CRC - PTX)	Pentoxiphylline	Phase 1	NCT06115174
Safety and Effectiveness Study of Pre-operative Artesunate in Stage II/III Colorectal Cancer (NeoART-V) (NeoART-V)	Artesunat	Phase 2	NCT03093129
Ursodiol, Combination Chemotherapy, and Bevacizumab in Treating Patients with Stage IV Colorectal Cancer	Ursodiol	Phase 3	NCT00873275
Effect of Silymarin in Metastatic Colorectal Cancer Patients	Silymarin	Phase 3	NCT05631041
Genistein in Treatment of Metastatic Colorectal Cancer	Genistein	Phase 1 Phase 2	NCT01985763
Resveratrol for Patients with Colon Cancer	Resveratrol	Phase 1	NCT00256334
Cancer Associated Thrombosis and Isoquercetin	Isoquercetin	Phase 2 Phase 3	NCT02195232

## 7 Conclusion

Over the past decade, the occurrence and fatality rates of CRC have shown a marked escalation, primarily attributed to the interplay of detrimental lifestyle patterns, dietary modifications, and additional contributing elements. Current investigations have highlighted the therapeutic potential of NDSMCs in the prevention and treatment of CRC, with this comprehensive review analyzing recent advancements in past 5 years. The analysis encompasses four critical biological mechanisms: gut microbiome regulation, ferroptosis modulation, and programmed cell death pathways (apoptosis and autophagy). Notably, phytochemical constituents including ginsenosides, berberine alkaloids, and curcuminoids have demonstrated promising outcomes in both preclinical studies and translational research, emerging as viable complementary approaches for CRC intervention strategies.

Nevertheless, certain limitations persist regarding the application of NDSMCs in nutritional supplementation and biomedical research. Primarily, NDSMCs are usually derived from natural resources such as animals, plants, and microorganisms, and their yields are easily affected by factors such as origin, season, and growth environment, and the scarcity of resources may limit large-scale research and application. Therefore, new sources should be actively explored, such as artificial breeding and cultivation raw materials, chemical synthesis and biosynthesis, etc. To enhance predictive accuracy regarding therapeutic potential and toxicological profiles while minimizing clinical trial complexities, integration of cutting-edge biotechnological tools becomes imperative. Genomic sequencing platforms, proteomic profiling systems, and metabolic pathway analyses, when synergized with computer-aided molecular modeling, could elucidate precise molecular targets and mechanistic pathways of NDSMCs. Many NDSMCs have problems such as low oral bioavailability, short half-life, fast metabolism, and unsatisfactory distribution *in vivo*, which affect their efficacy *in vivo*. The use of new formulation technologies, such as nanoparticles, liposomes, microcapsules, etc., to improve the pharmacokinetic properties of NDSMCs, improve bioavailability, prolong the time of action of drugs *in vivo*. Illustrating this trend, researchers have engineered various advanced curcumin delivery systems such as liposomal encapsulation, nanoparticle formulations, phospholipid complexes, and structural analogs to optimize its bioavailability. During preclinical evaluation phases, implementing rigorous safety assessment protocols-encompassing cytotoxicity analyses, chronic toxicity studies in animal models, and hypersensitivity assessments-is crucial for developing robust adverse event surveillance frameworks capable of promptly identifying and mitigating potential risks.
